# Predictive microRNAs for lymph node metastasis in endoscopically resectable submucosal colorectal cancer

**DOI:** 10.18632/oncotarget.8766

**Published:** 2016-04-16

**Authors:** Chan Kwon Jung, Seung-Hyun Jung, Seon-Hee Yim, Ji-Han Jung, Hyun Joo Choi, Won-Kyung Kang, Sung-Won Park, Seong-Taek Oh, Jun-Gi Kim, Sug Hyung Lee, Yeun-Jun Chung

**Affiliations:** ^1^ Department of Hospital Pathology, College of Medicine, The Catholic University of Korea, Seoul 06591, Republic of Korea; ^2^ Department of Microbiology, College of Medicine, The Catholic University of Korea, Seoul 06591, Republic of Korea; ^3^ Integrated Research Center for Genome Polymorphism, College of Medicine, The Catholic University of Korea, Seoul 06591, Republic of Korea; ^4^ Department of Surgery, College of Medicine, The Catholic University of Korea, Seoul 06591, Republic of Korea; ^5^ Department of Pathology, College of Medicine, The Catholic University of Korea, Seoul 06591, Republic of Korea; ^6^ Cancer Evolution Research Center, College of Medicine, The Catholic University of Korea, Seoul 06591, Republic of Korea

**Keywords:** endoscopically resectable colorectal cancer, microRNA, lymph node metastasis

## Abstract

Accurate prediction of regional lymph node metastasis (LNM) in endoscopically resected T1-stage colorectal cancers (CRCs) can reduce unnecessary surgeries. To identify miRNA markers that can predict LNM in T1-stage CRCs, the study was conducted in two phases; (I) miRNA classifier construction by miRNA-array and quantitative reverse transcription PCR (qRT-PCR) using 36 T1-stage CRC samples; (II) miRNA classifier validation in an independent set of 20 T1-stage CRC samples. The expression of potential downstream target genes of miRNAs was assessed by immunohistochemistry. In the discovery analysis by miRNA microarray, expression of 66 miRNAs were significantly different between LNM-positive and negative CRCs. After qRT-PCR validation, 11 miRNAs were consistently significant in the combined classifier construction set. Among them, miR-342-3p was the most significant one (*P*=4.3×10^−4^). Through logistic regression analysis, we developed a three-miRNA classifier (miR-342-3p, miR-361-3p, and miR-3621) for predicting LNM in T1-stage CRCs, yielding the area under the curve of 0.947 (94% sensitivity, 85% specificity and 89% accuracy). The discriminative ability of this system was consistently reliable in the independent validation set (83% sensitivity, 64% specificity and 70% of accuracy). Of the potential downstream targets of the three-miRNAs, expressions of E2F1, RAP2B, and AKT1 were significantly associated with LNM. In conclusion, this classifier can predict LNM more accurately than conventional pathologic criteria and our study results may be helpful to avoid unnecessary bowel surgery after endoscopic resection in early CRC.

## INTRODUCTION

Advances in colorectal cancer (CRC) screening programs have increased the chance of detecting malignant polyps at an early stage [1]. Most CRCs that are diagnosed at an early stage are reported to be curable [2]. Recently, endoscopic mucosal resection and endoscopic submucosal dissection have been widely used in the treatment of patients with T1-stage (submucosal invasion) CRC [2–5]. Although endoscopic treatment can achieve results oncologically equal to those of surgery in patients with T1-stage CRC with pathologically low-risk factors and complete resection margin [2–6], the effectiveness of guidelines for subsequent surgery versus surveillance after endoscopic resection of T1-stage CRCs remain controversial.

The rate of lymph node metastasis (LNM) is known to be associated with pathologic features such as depth of submucosal and lymphovascular invasion, histologic grading, and tumor budding [2–5, 7–10]. However, approximately 1.2% of low-risk cases of T1-stage CRCs without such risk factors develop regional LNM, which shows that these pathologic features are not enough to predict LNM [3]. As a result, concerns about local recurrence and LNM have led to surgical overtreatment in approximately 80% of patients who had undergone endoscopic resection of T1-stage CRC [11, 12]. If we can predict the likelihood of regional LNM more accurately for endoscopically resected T1-stage CRCs, the number of unnecessary additional surgeries can be reduced. For this, it is important to identify new markers which can help predicting LNM after endoscopic resection of T1-stage CRC.

There have been many studies to explore molecular genetic biomarkers for the prediction of LNM in early CRC [13]. One of the biomarkers is microRNA (miRNA) which is non-coding single-stranded RNA containing 19-25 nucleotides in length that regulates gene expression post-transcriptionally [14, 15]. A number of studies have reported that miRNAs are altered in colon adenoma and carcinoma and aberrant expressions of miRNAs have been shown to be involved in tumor cell growth, invasion, and metastasis of CRC [15, 16]. Therefore, miRNAs have been suggested as prognostic or predictive markers for survival and therapeutic outcomes of CRC [14–16]. However, little is known about the expression profiles of miRNAs and their clinical implications as predictive markers for LNM in T1-stage CRC.

The aim of the present study is therefore to identify specific miRNA markers that can predict LNM and facilitate accurate patient selection for subsequent surgery after endoscopic resection of T1-stage CRC.

## RESULTS

### Clinicopathologic characteristics of the classifier construction sets

**Figure 1:**
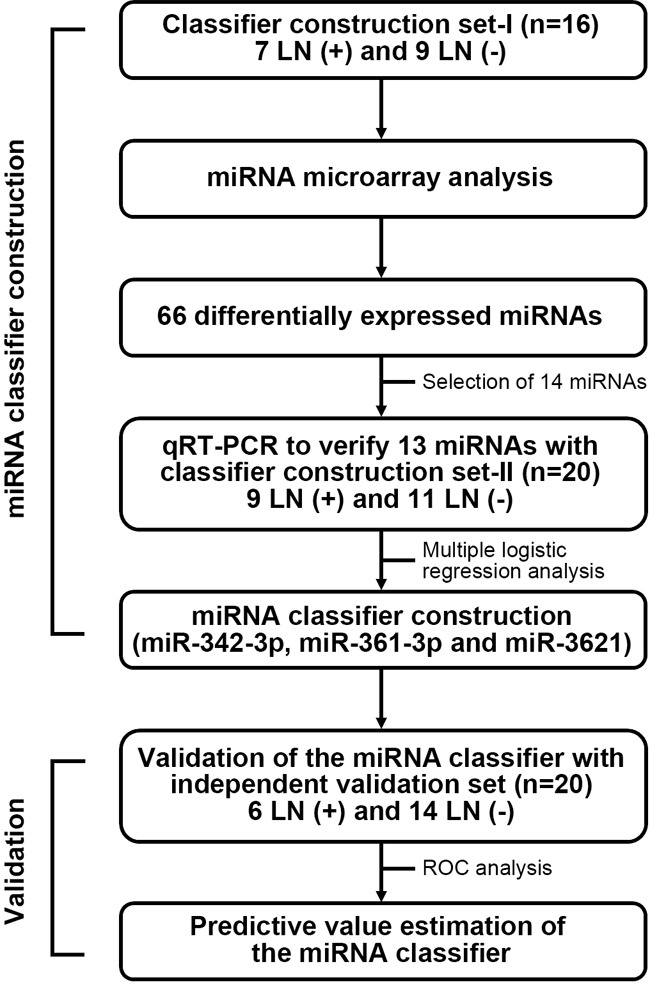
Study design and overall strategy for defining miRNA classifiers of LMN in T1-stage CRCs To identify miRNA markers that can predict LNM in T1-stage CRCs, the study was conducted in two phases; (I) miRNA classifier construction by miRNA-array and quantitative reverse transcription PCR (qRT-PCR) using 36 T1-stage CRC samples; (II) miRNA classifier validation in an independent set of 20 T1-stage CRC samples.

The study design and overall strategy for defining miRNA classifiers of LNM in T1-stage CRCs are illustrated in Figure 1. To identify predictive miRNA markers for LNM in early-stage CRC, we analyzed a total of 36 T1-stage CRC genomes (16 cases for classifier construction set-I and 20 cases for classifier construction set-II) (Table 1). The median ages of the LNM-positive and LNM-negative patients were 61 years and 56 years, respectively. All LNM-positive tumors (16/16) and 55% (11/20) of LNM-negative tumors had submucosal invasion (≥1,000 μm). Half of the LNM-positive tumors (8/16) and 15% (3/20) of the LNM-negative tumors had lymphatic invasion. Lymphatic invasion was significantly associated with LNM (*P*=0.034), whereas none of the other variables showed significant association with LNM. To validate the miRNA classifiers, we analyzed another 20 T1-stage CRC genomes as an independent validation set (Supplementary Table S1).

**Table 1:** Clinicopathologic features of the study subjects

Variables	Classifier construction set-I		Classifier construction set-II		Total classifier construction set		*P*-value
	LN metastasis		LN metastasis		LN metastasis		
	Negative (n=9)	Positive (n=7)	Negative (n=11)	Positive (n=9)	Negative (n=20)	Positive (n=16)	
Median age	55	60	61	62	56	61	0.223
Sex							
Male	5	3	9	6	14 (61%)	9 (39%)	0.393
Female	4	4	2	3	6 (46%)	7 (54%)	
Gross type							
Protruded pedunculated	1	2	4	3	5 (50%)	5 (50%)	
Protruded sessile	4	4	5	4	9 (53%)	8 (47%)	
Flat elevated	4	1	1	2	5 (62.5%)	3 (37.5%)	
Flat depressed	0	0	1	0	1 (100%)	0	
Location							
Right colon	1	1	6	2	7 (70%)	3 (30%)	0.514
Left colon	2	3	3	3	5 (45.5%)	6 (54.5%)	
Rectum	6	3	2	4	8 (53%)	7 (47%)	
Depth of invasion (μm)							
< 1000	3	0	6	0	9 (100%)	0	0.071
1000≤ or < 2000	2	3	1	4	3 (30%)	7 (70%)	
2000≤ or < 3000	3	4	3	4	6 (43%)	8 (57%)	
3000 ≤ or <4000	1	0	1	1	2 (67%)	1 (33%)	
Tumor depth							
sm1	6	5	7	5	13 (57%)	10 (44%)	
sm2	2	0	0	1	2 (67%)	1 (33%)	
Head invasion	1	0	2	0	3 (100%)	0	
Neck invasion	0	1	1	3	1 (20%)	4 (80%)	
Stalk invasion	0	1	1	0	1 (50%)	1 (50%)	
Tumor growth pattern							
Expanding	6	0	5	6	11 (65%)	6 (35%)	0.296
Infiltrating	3	7	6	3	9 (47%)	10 (53%)	
Histologic differentiation							
Well	2	0	6	3	8 (73%)	3 (27%)	0.345
Moderately	7	7	4	6	11 (46%)	13 (54%)	
Poorly	0	0	1	0	1 (100%)	0	
Tumor budding							
Low	6	4	9	6	15 (60%)	10 (40%)	0.483
High	3	3	2	3	5 (46%)	6 (55%)	
Lymphatic invasion							
Absent	8	4	9	4	17 (68%)	8 (32%)	0.034
Present	1	3	2	5	3 (27%)	8 (73%)	
Initial endoscopic removal							
Yes	4	6	5	4	9 (47%)	10 (53%)	0.296
No	5	1	6	5	11 (65%)	6 (35%)	

### MiRNA expression profiles associated with lymph node metastasis

**Figure 2:**
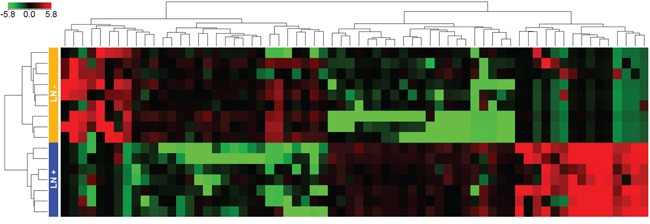
Unsupervised hierarchical clustering analysis of the 66 differentially expressed miRNAs Seven LNM-positive and nine LMN-negative CRCs were clearly clustered. Green and red represent the downregulated and upregulated miRNAs, respectively. Blue and orange boxes represent the LNM-positive and LNM-negative CRCs, respectively.

In the classifier construction set-I, we examined global miRNA expression profiles of 16 T1-stage CRCs (7 LNM-positive and 9 LNM-negative tumors) using miRNA array analysis. A total of 66 miRNAs were found to be differentially expressed between the two groups (Supplementary Table S2). Among them, 36 miRNAs were upregulated and 30 were downregulated in LNM-positive CRCs compared with LNM-negative CRCs. Unsupervised hierarchical clustering analysis of the differentially expressed miRNAs showed a clear discrepancy between the patterns in LNM-positive and -negative T1-stage CRCs (Figure 2).

### Selection of miRNA candidate and qRT-PCR validation

From the 66 differentially expressed miRNAs, we selected 13 miRNAs as candidate markers of LNM in T1-stage CRCs according to the selection criteria as follows; we excluded the miRNAs showing >50 fold changes; we selected the miRNAs which had been reported to be correlated with tumorigenesis or LNM (10 downregulated: miR-342-3p, miR-140-3p, miR-200b-5p, miR-150-5p, miR-28-5p, miR185-5p, miR-361-3p, miR-192-3p, miR-30a-5p, and miR-195-5p; and 3 upregulated: miR-3621, miR-1287, and miR-3132). Details are available in Supplementary Table S3. For the validation of their array-based expression profiles, we first performed qRT-PCR with the same CRC RNAs used in the classifier-construction set-I. Up- or downregulated expression profiles from the arrays were generally consistent with those defined by qRT-PCR (Supplementary Table S3). We then performed independent replication experiments for the 13 miRNAs using the classifier construction set-II (20 T1-stage CRCs; 9 LNM-positives and 11 LNM-negatives). In the replication experiments, 12 of the 13 candidates showed the same trend of up- or downregulation as in the miRNA array analysis. When we combined the data from the set-I and -II (a total of 36 T1-stage CRCs), the differences in expression of 11 miRNAs (8 downregulated: miR-342-3p, miR-140-3p, miR-200b-5p, miR-150-5p, miR185-5p, miR-361-3p, miR-192-3p, and miR-195-5p; and 3 upregulated: miR-3621, miR-1287, and miR-3132) were consistently significant. Of the 11 miRNAs, the level of significance was the highest in miR-342-3p (*P*=4.3×10^−4^) (Supplementary Table S3).

### Development of miRNA classifier system for predicting LNM

**Figure 3:**
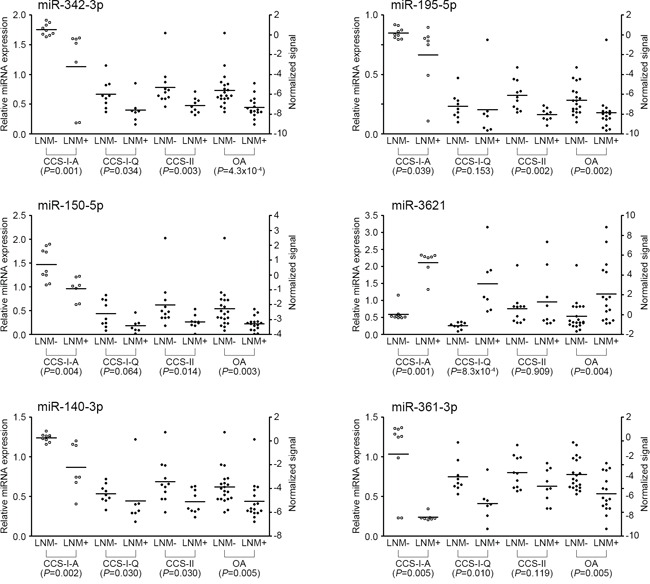
Expression levels of six miRNAs measured by qRT-PCR Relative miRNA expression level of each miRNA was normalized to *RNU6B*. Statistical significance was calculated using Mann-Whitney U test between LNM-positive and -negative CRCs. Left side Y-axis represents fold changes based on the normal colorectal tissue as the calibrator. Right side Y-axis represents normalized signal intensities on a log2 scale. Open circles represent microarray experiment and black filled circles represent qRT-PCR experiment. CCS-I-A, Array-expression data from the classifier construction set-I; CCS-I-Q, qRT-PCR data from the classifier construction set-I; CCS-II, qRT-PCR data from the classifier construction set-II; OA, overall qRT-PCR data from the classifier construction set-I and II; LNM, lymph node metastasis.

**Figure 4:**
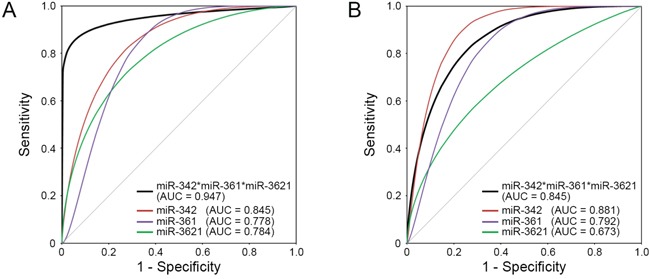
ROC curves for the three individual miRNAs and the ‘three-miRNA classifier’ The three-miRNA classifier (black line) shows the best sensitivity and specificity compared with individual miRNAs (red, purple, and green lines) in the **A.** combined classifier construction set and **B.** independent validation set. AUC, area under the curve.

To improve the applicability of the differentially expressed miRNAs for predicting LNM in T1-stage CRCs, we chose the top six significant targets (*P* <0.005 in the combined classifier construction set: downregulated miR-342-3p, miR-195-5p, miR-150-5p, miR140-3p and miR-361-3p and upregulated miR-3621) from the 11 candidate miRNAs to select the miRNA classifiers. Figure 3 illustrates the expression profiles of the top six significant miRNAs (*P*<0.005) identified by microarray and qRT-PCR. We then performed the ROC analysis for the six miRNAs. The ROC curves showed AUC values of 0.845, 0.806, 0.791, 0.784, 0.778 and 0.778 for miR-342-3p, miR-195-5p, miR-150-5p, miR3621, miR140-3p and miR-361-3p, respectively (Table 2). To increase the prediction efficiency, we performed multiple logistic regression analysis with backward selection method as described previously [17]. Through the logistic regression analysis, we selected the classifier comprised of the three miRNAs; Logit (P) = 7.37 - (9.95*miR-342-3p) + (4.241*miR-3621) - (8.135*miR361-3p) (Supplementary Table S4). This miRNA classifier (downregulation of miR-342-3p and miR-361-3p, and upregulation of miR-3621) had the best sensitivity and specificity; AUC of 0.947 (95% CI: 0.872-1.000), 94% sensitivity, 85% specificity, and 89% accuracy (Table 2, Figure 4A).

**Table 2:** Test performance of top six miRNAs and three-miRNA classifier for predicting LNM

miRNA	AUC	95% CI	Sensitivity	Specificity	Accuracy
miR-342-3p	0.845	0.711-0.979	90%	69%	78%
miR-195-5p	0.806	0.655-0.958	75%	81%	78%
miR-150-5p	0.791	0.643-0.938	75%	81%	78%
miR-3621	0.784	0.634-0.934	88%	60%	72%
miR-140-3p	0.778	0.617-0.939	90%	69%	78%
miR-361-3p	0.778	0.615-0.942	85%	69%	76%
^1)^3-miRNA classifier (miR-342-3p, miR-3621 and miR-361-3p)	0.947	0.872-1.000	94%	85%	89%

1) Logit (P) = 7.37 - (9.95*miR-342-3p) + (4.241*miR-3621) - (8.135*miR-361-3p)

### Validation of the miRNA classifier

After developing the miRNA classifier, we validated this system with an independent set of T1-stage CRCs with and without LNM (20 CRCs; 6 LNM-positive and 14 LNM-negative). In the independent set, the discriminative ability of this classifier system was found to be highly reliable: AUC of 0.845 (95% CI: 0.670-1.000), 83% sensitivity, 64% specificity and 70% of accuracy (Figure 4B).

### Gene ontology and pathway analysis of target genes of the candidate miRNAs

To gain insight into the functions of the three miRNAs which consist of the classifier, their target genes were predicted using miRWalk database [18]. DAVID analysis showed that the target genes of those miRNAs were significantly associated with important tumorigenesis- and metastasis-related pathways (Supplementary Table S5). These signaling pathways included 'Pathways in cancer', 'Focal adhesion', 'Wnt signaling', 'MAPK signaling' and 'Colorectal cancer', which have been demonstrated to participate in tumorigenesis, tumor progression, and recurrence. The 'mTOR signaling' pathway was significantly enriched in miR-342-3p and miR-361-3p. 'Apoptosis' pathway was significantly enriched in miR361-3p and miR-3621. Supplementary Figure S1 shows the top five pathways for each miRNA. Gene ontology analysis also showed that tumorigenesis- and metastasis-related ontologies were regulated by the three miRNAs (Supplementary Table S6).

### Immunohistochemical analysis of predicted targets of the three miRNAs

**Figure 5:**
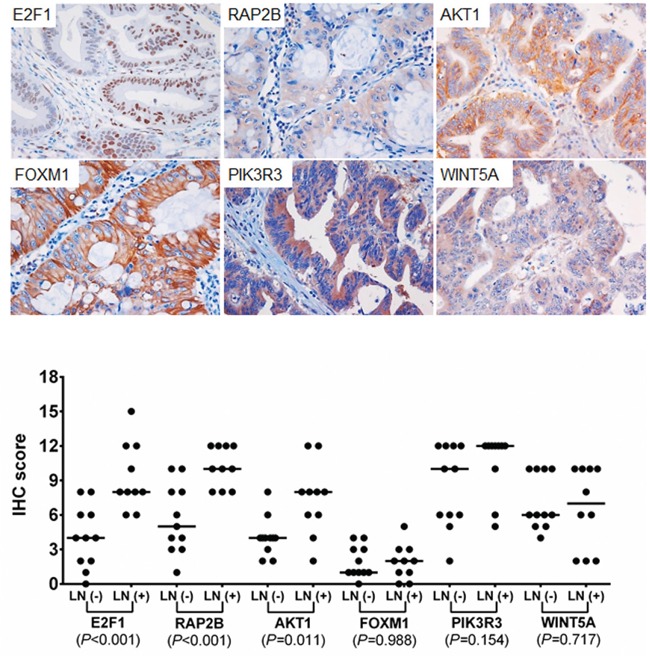
Immunohistochemical staining for E2F1, RAP2B, FOXM1, PIK3R3, AKT1, and WINT5A Upper plots represent the examples of positive immunohisctochemcial (IHC) staining of each target. IHC scores were analyzed using Mann-Whitney U test between LNM-positive and –negative CRCs for each marker.

**Figure 6:**
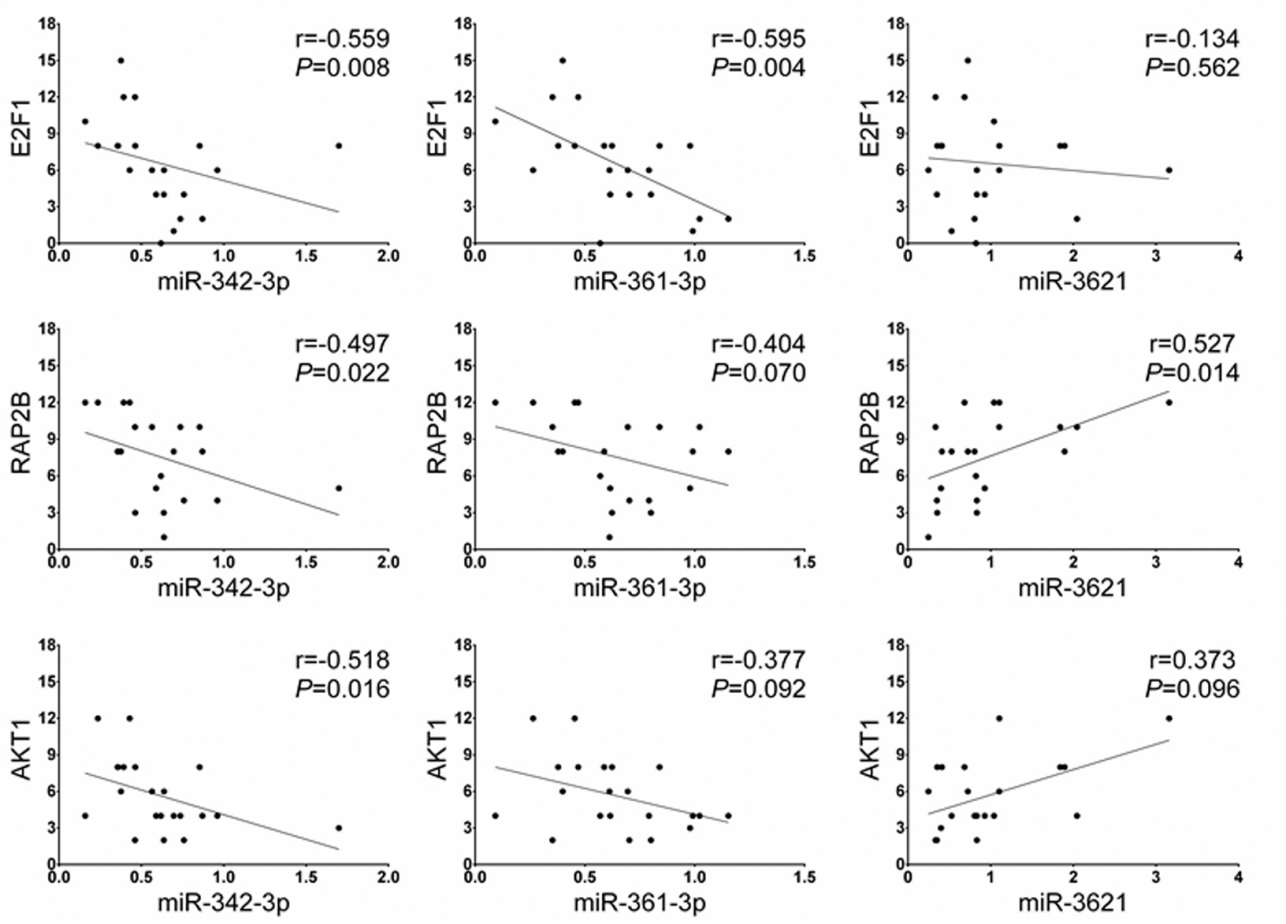
Spearman's rho correlation between the miRNA and downstream target protein expression levels The expression level of miR-342-3p shows a negative correlation with the protein levels of E2F1, RAP2B, and AKT1. The expression level of miR-361-3p is negatively correlated with E2F1 expression and miR-3621 is positively correlated with RAP2B expression.

We assessed whether the expression of the potential target genes of three miRNAs are different between LNM-positives and negatives by immunohistochemical analysis. Six potential target genes of miR-342-3p, miR-361-3p, and miR-3621 (*E2F1*, *RAP2B*, *FOXM1*, *PIK3R3*, *AKT1* and *WINT5A*) were selected based on union search of three miRNA prediction algorithms (miRWalk, miRanda, and TargetScan) using miRWalk database as well as a literature search [19–29]. Immunohistochemistry was performed for the six target proteins with 10 LNM-positive and 11 LNM-negative T1-stage CRCs (Figure 5). Immunohistochemical scores of E2F1, RAP2B, and AKT1 were significantly higher in the LNM-positive group than in LNM-negative group (Figure 5), while the expression of FOXM1, PIK3R3, and WINT5A was not associated with LNM status. We further analyzed the correlations between the expression levels of these proteins and miRNAs. The expression level of miR-342-3p was inversely correlated with the expression levels of E2F1 (r=−0.559; *P*=0.008), RAP2B (r=−0.497; *P*=0.022), and AKT1 (r=−0.518; *P*=0.016) (Figure 6). Also, miR-361-3p showed an inverse correlation with E2F1 expression (r=−0.595; *P*=0.004) and miR-3621 was positively correlated with RAP2B expression (r=0.527; *P*=0.014).

## DISCUSSION

Patients with T1-stage CRC with any of the following risk factors have been considered to be at high risk for LNM even after complete endoscopic resection; poorly differentiated adenocarcinoma, signet ring cell or mucinous carcinoma, presence of vascular or lymphatic invasion, depth of submucosal invasion ≥1,000 μm, or ≥5 tumor budding foci in the invasive front [7, 10]. High-risk patients are usually referred for subsequent radical surgery [7, 10]. In a recent meta-analysis, the LNM rate was 14.6% (95% CI = 12.4-17.0) in T1-stage CRC with submucosal invasion ≥1,000 μm, compared with 1.90% (95% CI = 0.52-4.78) in those with submucosal invasion <1,000 μm [30]. In our study, all LNM-positive CRCs had *submucosal invasion ≥1,000 μm;* however, other risk factors such as poorly differentiated adenocarcinoma, ≥5 tumor budding foci, and lymphatic invasion were also found in LNM-negative CRCs. Previous studies and our data suggest that pathologic risk criteria can predict LNM with high sensitivity, but with relatively low specificity, which may lead to surgical overtreatment [7, 10, 31].

To develop miRNA markers that can predict LNM-positive tumors among T1-stage CRCs, we first examined the miRNA expression profiles by miRNA array analysis using archival formalin-fixed paraffin-embedded (FFPE) specimens of primary T1-stage CRCs. The differentially expressed miRNAs identified in the classifier construction set included known LNM-related miRNAs in CRCs such as miR-99b, miR-139, miR-195, miR-199b and miR-342 [32–34]. In addition, a number of miRNAs previously reported to be differentially expressed in CRCs were also identified in this study (miR-101, miR-130a, miR-192, miR-214, and miR-230a), whose expression patterns were consistent with those from the previous reports [35–38]. These data support the reliability of our miRNA screening.

Among the top six significant miRNAs from the combined analysis, miR-342, which targets the *DNMT1* and inhibits tumor cell proliferation or invasion, has been suggested as a tumor suppressor in CRC [39]. The expression of miR-342 was reported to be significantly downregulated in all stages of CRC compared with adjacent normal tissues [39]. In our study, the expression of miR-342-3p was the most significantly different one between the LNM-positive and -negative T1-CRCs (*P*=4.3×10^−4^). Regarding miR-361, miR-361-5p has been suggested to play a tumor suppressor role in diverse solid tumors including CRC [40, 41]. Although, the role of miR-361-3p has not been studied in CRC, it has been reported to be downregulated in lung and prostate cancers [42, 43]. Downregulation of miR-195 was reported to be associated with LNM and poor prognosis in CRC [34]. The expression of miR-150 was also reported to decrease with the progression from normal colorectal mucosa to adenoma to CRC and was frequently downregulated in CRCs, which was associated with poor therapeutic outcome and prognosis [44–46]. The expression of miR-140 was reported to be downregulated in CRCs and its overexpression suppressed colon cancer cell proliferation by inducing p53 and p21 expression or by reducing histone deacetylase 4 expression [47]. Although the biological implications of miR-361-3p and miR-3621 have not been studied in CRC, pathway analysis revealed that their target genes may play a role in colorectal tumorigenesis and metastasis. However, to facilitate the application of the miRNA markers, we developed three-miRNA based classifier system that could predict LNM in T1-stage CRCs with 94% sensitivity, 85% specificity and 89% accuracy.

When we examined the expression of the potential downstream target genes of the miRNAs according to LNM status, the expressions of E2F1, RAP2B, and AKT1 were significantly associated with LNM and their expression levels were inversely correlated with the miRNA levels. E2F1 overexpression was reported to be correlated with LNM and distant metastasis in CRC [22]. In addition, E2F1 and RAP2B were reported to promote progression and metastasis of lung cancer and their expressions was downregulated by miR-342-3p in lung cancer [26, 28]. We also showed that miR-3621 was upregulated in LNM-positive CRCs and its expression level was positively correlated with RAP2B expression. Therefore, downregulation of miR-342-3p and miR-361-3p, and upregulation of miR-3621 may promote LNM of T1-stage CRC through upregulation of E2F1 or RAP2B expression.

Our study is distinct from previous studies in several aspects. First, to identify miRNA markers that can predict LNM specifically in early-stage CRCs, we compared the miRNA profiles in T1-stage CRCs by the presence of LNM instead of simply comparing paired tumor and normal tissues. Most of the previous studies just identified differentially expressed miRNAs in CRC and some of those had prognostic implications. Our findings, although the expression profiles of the six miRNAs appeared to be similar or consistent with those of previous studies, are LNM-specific in early-stage colorectal carcinogenesis, not about colon carcinogenesis in general. Second, to improve the clinical applicability of the miRNA markers, we developed a miRNA-based classifier panel rather than just suggesting each miRNA as predictor. Indeed, the predictive value of the three-miRNA panel was superior to that of any individual miRNAs, which seems to be natural since cancer is a multigenic disorder. The discriminative ability of this system was consistently reliable in the independent validation set.

In summary, we developed a ‘three-miRNA classifier’ for predicting LNM in T1-stage CRCs. This classifier was proven to predict LNM more accurately than conventional pathologic criteria. This classifier and our study results may be helpful to avoid unnecessary bowel surgery after curative endoscopic resection in patients with early CRC.

## MATERIALS AND METHODS

### Patients

For the classifier construction, we retrospectively reviewed the records of patients with T1-stage CRC who underwent curative surgical resection with lymph node dissection as first- or second-line treatment at Seoul St. Mary's Hospital, the Catholic University of Korea, between January 2007 and December 2012. Inclusion criteria were T1-stage CRC with submucosal invasion less than 4,000 μm from the muscularis mucosa and of less than two-thirds of the submucosa. Patients who meet any of the following criteria were not eligible for the study; invasion of the lower third of the submucosa; presence of synchronous CRC or other malignancy; no surgical removal of regional lymph nodes as first- or second-line treatment. A total of 36 patients with submucosal invasive CRC were enrolled in the study (Table 1). Among these patients, 19 were initially treated with endoscopic resection and then underwent additional curative surgery and 17 were primarily treated by surgery with lymph node dissection. Subjects were randomly divided into two sets (classifier construction set-I and classifier construction set-II). The classifier construction set-I was composed of seven LNM-positive and nine LNM-negative CRCs and the independent classifier construction set-II was composed of nine LNM-positive and eleven LNM-negative CRCs. None of the patients received preoperative chemotherapy or radiation therapy. For the validation of the miRNA classifier, 20 T1-stage CTCs with and without LNM (6 LNM-positive and 14 LNM-negative) were enrolled independently. The validation set was obtained from Seoul St. Mary's Hospital between January 2013 and December 2014 using the same enrolment criteria. This study was approved by the Institutional Review Board of the Catholic University Medical College of Korea (MC13SNS10023).

### Pathologic evaluation

The gross appearances of CRCs were classified into protruded pedunculated, protruded sessile, flat elevated, and flat depressed. Histologic diagnosis and grading were based on World Health Organization classification [48]. The depth of submucosal invasion was measured according to the 2010 Japanese guidelines [7]. When the muscularis mucosa was identified or presumed, the depth of submucosal invasion was measured from the lower border of muscularis mucosae to the deepest invasive margin regardless of the gross type. When the muscularis mucosae were not seen by the tumor invasion, the depth was measured from the surface of the tumor to the deepest invasive margin regardless of the gross type. In the pedunculated polyps, when the submucosal invasion was identified below the baseline between the tumor head and the stalk and tangled muscularis mucosae were found, the depth of submucosal invasion was measured from the baseline to the deepest portion of invasion. When the submucosal invasion was limited to the head of the pedunculated polyp, the depth of submucosal invasion was considered to be 0 μm. The submucosal invasion was also semi-quantitatively divided into tertiles (sm1, sm2, and sm3) based on the depth of tumor invasion. As endoscopically resected specimens do not include the full-thickness layer of submucosal tissue, the level of submucosal invasion in the endoscopically resected specimens was estimated through combining histologic features of both endoscopic and surgical resection specimens. Tumor budding was defined as the presence of isolated cell or tumor cell nests comprising 1-4 cells at the invasive front of the cancer. The budding was classified into low grade (1-4 foci) and high grade (5 or more foci) based on the number of tumor buds counted at 200-fold magnification [7].

### RNA preparation

FFPE tissue sections (thickness 15 μm) were deparaffinized with xylene, washed with ethanol and then dried. Tumor cell rich areas (>70% of tumor cells) were selected and microdissected under a microscope. Total RNA was extracted using the RecoverAll™ Total Nucleic Acid Isolation Kit for FFPE (Life technologies, Carlsbad, CA). The total RNA concentrations of the samples were measured using a NanoDrop ND-1000 spectrophotometer (Thermo Scientific, Wilmington, DE). RNA quality was measured using Agilent RNA 6000 Nano Kit with an Agilent 2100 Bioanalyzer (Agilent Technologies, Santa Clara, CA).

### MiRNA expression profiling

Agilent Human miRNA Microarray Kit (Release 16.0, Agilent technologies), which contains the probes for 1,205 human miRNAs and 144 human viral miRNAs, was used for miRNA expression profiling. MiRNA array experiments were conducted according to the manufacturer's instructions. In brief, 100 ng of total RNA from each sample was dephosphorylated and ligated with pCp-Cy3 dye. After purifying the labeled RNA using Micro Bio-Spin 6 column (Bio-Rad, Hercules, CA), labeled RNA was applied on the miRNA array with a hybridization buffer. Array slides were incubated for 20 hours at 55°C. After washing and scanning the arrays, images were analyzed with Feature Extraction 10.7.3.1 software (Agilent technologies). Data quality was assessed using the Agilent's microRNA Spike-In kit and all samples were passed the Spike-In QC criteria (LabelingSpike-InSignal > 2.5 and HybSpike-InSignal > 2.5). The miRNA microarray data is available in NCBI Gene Expression omnibus (GSE70574).

### Data analysis for miRNA microarray

The miRNA array data was processed by quantile normalization, followed by log2 transformation. The spots called ‘absent’ by Agilent's Feature Extraction software were discarded. The unpaired Mann-Whitney test was used to identify significant differences in expressed miRNA between LNM-positive and -negative CRC samples. The Benjamini-Hochberg false discovery rate (FDR) method was used for multiple comparison correction. MiRNAs with FDR<0.1 and log fold change >3 were considered to be potentially significant and included in further independent replication experiments to validate. All data analysis processing was conducted using GeneSpring 12.6 software (Agilent technologies).

### Quantitative reverse transcription PCR validation and replication

To validate and replicate the miRNAs that may be involved in LNM, quantitative reverse transcription PCR (qRT-PCR) was performed using the TaqMan MicroRNA Assay (miR-342-3p, #002260; miR-195-5p, #000494; miR-150-5p, #000473; miR-140-3p, #002234; miR-361-3p, #002116; miR-192-3p, #002272; miR-200b-5p, #002274; miR-185-5p, #002271; miR-30a-5p, #000417; miR-28-5p, #000411; miR-3621, #463091_mat; miR-1287, #002828; miR-3132, #243376_mat; *RNU6B*, #001093) and the ViiA7 system (Life Technologies) according to the manufacturer's protocol. In Brief, 10ng of total RNA was converted to first-strand cDNA with miRNA-specific primers using TaqMan MicroRNA Reverse Transcription Kit (#4366596, Life Technologies), followed by real-time PCR with TaqMan Probes. Total RNA from normal colorectal tissue (#AM7986, Life Technologies) was used as calibrator. The expression level of each miRNA target was defined as 2^−ΔΔCt^, where ΔCt is the difference in threshold cycles for the sample in question, normalized against the endogenous gene (*RNU6B*) and expressed relative to the value obtained by the calibrator (individual/calibrator) as described elsewhere [49]. All PCR reactions for each sample were carried out in triplicate.

### Gene ontology and pathway analysis

Target genes of the miRNAs were predicted using miRWalk database (http://zmf.umm.uni-heidelberg.de/apps/zmf/mirwalk2/index.html) with default parameters [18]. We observed the pathway-level relationship of target genes of miRNAs using the database for annotation, visualization and integrated discovery (DAVID) v6.7 bioinformatics tools (http://david.abcc.ncifcrf.gov/) [50]. Pathway analysis was used to find out the significant pathways of the predicted target genes according to Kyoto Encyclopedia of Genes and Genomes (KEGG) pathways in the DAVID.

### Immunohistochemistry

FFPE tissue sections (thickness 4 μm) were deparffinized with xylene and rehydrated with a graded series of ethanol. Sections were treated in an electric pressure cooker (Couisinart, Cell Marque, Rocklin, CA) at high pressure in 0.01 M sodium citrate buffer (pH 6.0) for 20 minutes for antigen retrieval. Endogenous peroxidase was quenched with 3% hydrogen peroxide diluted methanol for 15 minutes. Sections were incubated with the primary antibodies for 1 hour at room temperature. The following antibodies were used: anti-E2F1 (1:50; mouse monoclonal; SantaCruz, Dallas, TX), anti-RAP2B (1:50; mouse monoclonal; Abcam; Cambridge, MA), anti-AKT1 (1:100; rabbit polyclonal; Novus, Littleton, CO), anti-FOXM1 (1:50; rabbit polyclonal; Abcam), anti-PIK3R2 (1:20; rabbit polyclonal; Novus), WINT5A (1:50; rabbit polyclonal; Novus). Negative controls included substitution of the specific primary antibodies with an equivalent concentration of the mouse or rabbit serum. The immunoreaction was amplified with Polink-1 HRP Detection System for Broad Spectrum (GBI Labs, Mukilteo, WA, USA), following the manufacturer's directions, and visualized with 3,3′-diaminobenzidine (DAB). The slides were counterstained with hematoxylin.

Immunohistochemical staining was scored semiquantitatively by multiplying scores for staining intensity (0, absent; 1, weak; 2, moderate; or 3, strong) and percentage of stained tumor cells (0, 0%; 1, 1-10%; 2, 11-25%; 3, 26-50%; 4, 51-75%; 5, 76-90%; and 6, 91-100%). Scores ranged from 0 to 18.

### Statistical analysis

Pearson's chi-squared test or Fisher's exact test was used to analyze the associations between the categorical clinicopathologic variables. The Mann-Whitney U test or Kruskal-Wallis test was used for continuous clinicopathologic variables. Spearman's rank correlation coefficient was used to identify the strength of a relationship between the expression levels of miRNAs and the protein expression levels of their targets. The receiver operating characteristic (ROC) curve and area under curve (AUC) were used to assess the predictive value of miRNA markers for LNM. Multiple logistic regression analysis was used to generate miRNA classifiers. Statistical analyses were performed using SPSS (version 21, Chicago, IL). GraphPad Prism software (version 6, La Jolla, CA) was used to create graphs. All *P* values <0.05 were considered significant.

## SUPPLEMENTARY FIGURES AND TABLES










