# Supplementary material for: Predictive microRNAs for lymph node metastasis in endoscopically resectable submucosal colorectal cancer
Source: Oncotarget. 2016 Apr 16;7(22):32902–15. doi: 10.18632/oncotarget.8766 (PMC5078061; doi:10.18632/oncotarget.8766)
Supplement: Supplementary file 1 [file oncotarget-07-32902-s001.pdf]

Predictive microRNAs for lymph node metastasis in endoscopically resectable submucosal colorectal cancer

SUPPLEMENTARY DATA

REFERENCES

1. Wang H, Wu J, Meng X, Ying X, Zuo Y, Liu R, Pan Z, Kang T, Huang W. MicroRNA-342 inhibits colorectal cancer cell proliferation and invasion by directly targeting DNA methyltransferase 1. Carcinogenesis. 2011; 32:1033-1042.
2. Ding J, Huang S, Wang Y, Tian Q, Zha R, Shi H, Wang Q, Ge C, Chen T, Zhao Y, Liang L, Li J, He X. Genome-wide screening reveals that miR-195 targets the TNF-alpha/NF-kappaB pathway by down-regulating IkappaB kinase alpha and TAB3 in hepatocellular carcinoma. Hepatology. 2013; 58:654-666.
3. Wang WH, Chen J, Zhao F, Zhang BR, Yu HS, Jin HY, Dai JH. MiR-150-5p suppresses colorectal cancer cell migration and invasion through targeting MUC4. Asian Pacific journal of cancer prevention: APJCP. 2014; 15:6269-6273.
4. Piepoli A, Tavano F, Copetti M, Mazza T, Palumbo O, Panza A, di Mola FF, Pazienza V, Mazzoccoli G, Biscaglia G, Gentile A, Mastrodonato N, Carella M, Pellegrini F, di Sebastiano P, Andriulli A. Mirna expression profiles identify drivers in colorectal and pancreatic cancers. PloS one. 2012; 7:e33663.
5. Roth C, Stuckrath I, Pantel K, Izbicki JR, Tachezy M, Schwarzenbach H. Low levels of cell-free circulating miR-361-3p and miR-625* as blood-based markers for discriminating malignant from benign lung tumors. PloS one. 2012; 7:e38248.
6. Geng L, Chaudhuri A, Talmon G, Wisecarver JL, Are C, Brattain M, Wang J. MicroRNA-192 suppresses liver metastasis of colon cancer. Oncogene. 2014; 33:5332-5340.
7. Paterson EL, Kazenwadel J, Bert AG, Khew-Goodall Y, Ruszkiewicz A, Goodall GJ. Down-regulation of the miRNA-200 family at the invasive front of colorectal cancers with degraded basement membrane indicates EMT is involved in cancer progression. Neoplasia. 2013; 15:180-191.
8. Wang B, Li J, Sun M, Sun L, Zhang X. miRNA expression in breast cancer varies with lymph node metastasis and other clinicopathologic features. IUBMB Life. 2014; 66:371-377.
9. Zhang N, Wang X, Huo Q, Sun M, Cai C, Liu Z, Hu G, Yang Q. MicroRNA-30a suppresses breast tumor growth and metastasis by targeting metadherin. Oncogene. 2014; 33:3119-3128.
10. Almeida MI, Nicoloso MS, Zeng L, Ivan C, Spizzo R, Gafa R, Xiao L, Zhang X, Vannini I, Fanini F, Fabbri M, Lanza G, Reis RM, Zweidler-McKay PA and Calin GA. Strand-specific miR-28-5p and miR-28-3p have distinct effects in colorectal cancer cells. Gastroenterology. 2012; 142:886-896 e889.
11. Miao Y, Li J, Qiu X, Li Y, Wang Z, Luan Y. miR-27a regulates the self renewal of the H446 small cell lung cancer cell line in vitro. Oncol Rep. 2013; 29:161-168.
12. Guo L, Zhao Y, Yang S, Cai M, Wu Q, Chen F. Genome-wide screen for aberrantly expressed miRNAs reveals miRNA profile signature in breast cancer. Mol Biol Rep. 2013; 40:2175-2186.
13. Stark MS, Tyagi S, Nancarrow DJ, Boyle GM, Cook AL, Whiteman DC, Parsons PG, Schmidt C, Sturm RA, Hayward NK. Characterization of the Melanoma miRNAome by Deep Sequencing. PloS one. 2010; 5:e9685.

Supplementary Table S1: Clinicopathologic features of the independent validation set

Variables	Negative (n=14)	Positive (n=6)
Median age	57	61
Sex		
Male	9	4
Female	5	2
Gross type		
Protruded pedunculated	8	1
Protruded sessile	3	5
Flat elevated	3	0
Flat depressed	0	0
Location		
Right colon	6	0
Left colon	3	5
Rectum	5	1
Depth of invasion (μm)		
< 1000	5	1
1000≤ or < 2000	8	4
2000≤ or < 3000	1	1
3000 ≤ or <4000	0	0
Tumor depth		
sm1	13	4
sm2	0	0
Head invasion	0	0
Neck invasion	0	2
Stalk invasion	1	0
Tumor growth pattern		
Expanding	7	1
Infiltrating	7	5
Histologic differentiation		
Well	4	2
Moderately	10	4
Poorly	0	0
Tumor budding		
Low	13	4
High	1	2
Lymphatic invasion		
Absent	0	4
Present	14	2
Initial endoscopic removal		
Yes	4	1
No	10	4

Supplementary Table S2: List of the 66 differentially expressed miRNAs between LNM-positive and LNM-negative CRCs in classifier construction set I

See Supplementary File 1

Supplementary Table S3: Selection and replication of the 13 miRNAs

See Supplementary File 2

Supplementary Table S4: Logistic regression analysis with backward selection method

Variable	B	S.E.	Wald	df	Sig.	Exp(B)	95%CI for EXP (B)	
							Lower	Upper
miR-342-3p	-9.950	5.901	2.843	1	0.092	0.000	0.000	5.031
miR-361-3p	-8.135	5.191	2.456	1	0.117	0.000	0.000	7.686
miR-3621	4.241	1.741	5.934	1	0.015	69.486	2.290	2108.186
Constant	7.370	3.365	4.796	1	0.029	1587.166		

Supplementary Table S5: Top twenty pathways significantly enriched with the predicted target genes of the three miRNAs

See Supplementary File 3

Supplementary Table S6: Top twenty gene ontology sets significantly enriched with the predicted target genes of the three miRNAs

See Supplementary File 4

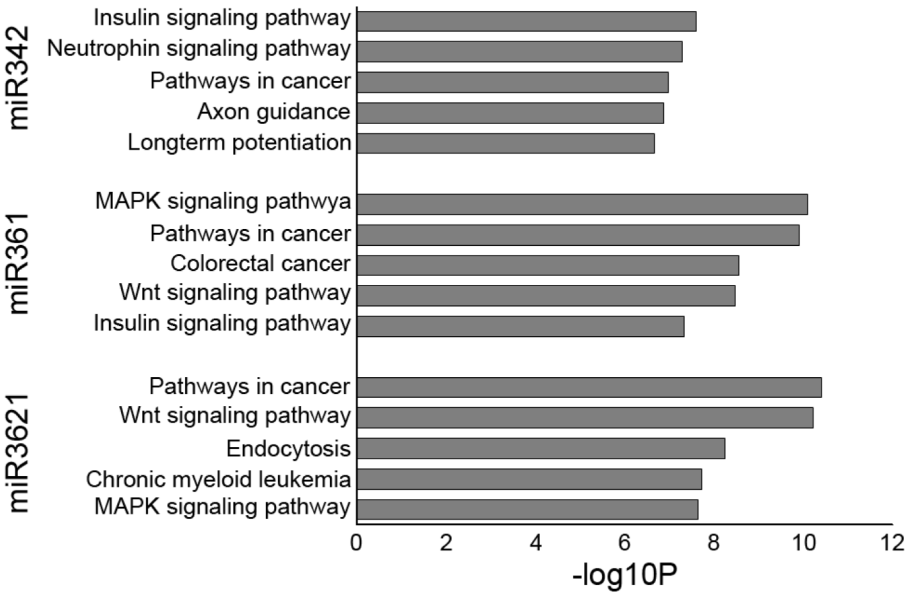

Supplementary Figure S1: Pathway analysis for the three individual miRNAs. Top five pathways significantly enriched with the predicted target genes of the three miRNAs are shown. Target genes of the three miRNAs were predicted using miRWalk database. The database for annotation, visualization and integrated discovery (DAVID) v6.7 bioinformatics tool was used to observe the pathway-level relationship. X axis represents the significance level.
