# Supplementary material for: Predictive microRNAs for lymph node metastasis in endoscopically resectable submucosal colorectal cancer
Source: Oncotarget. 2016 Apr 16;7(22):32902–15. doi: 10.18632/oncotarget.8766 (PMC5078061; doi:10.18632/oncotarget.8766)
Supplement: Supplementary file 2 [file oncotarget-07-32902-s002.pdf]

Table S2. List of the 66 differentially expressed miRNAs between LNM-positive and LNM-negative CRCs in classifier construction set I

Up-regulated miRNAs				Down-regulated miRNAs			
miRNA	*P*-value	FDR	Fold	miRNA	*P*-value	FDR	Fold
hsa-miR-3666	0.0008	0.0146	68.5	hsa-miR-342-3p	0.0009	0.0146	13.4
hsa-miR-4314	0.0008	0.0146	3.6	hsa-miR-4317	0.0009	0.0146	69.9
hsa-miR-H16	0.0008	0.0146	13.2	hsa-miR-3607-3p	0.0012	0.0167	6.4
hsa-miR-B20	0.0009	0.0146	202.3	hsa-miR-140-3p	0.0018	0.0167	5.6
hsa-miR-3197	0.0009	0.0146	179.7	hsa-miR-375	0.0018	0.0167	8.6
hsa-miR-3621	0.0009	0.0146	37.0	hsa-miR-374b-5p	0.0021	0.0168	3.9
hsa-miR-602	0.0009	0.0146	125.5	hsa-miR-200b-5p	0.0025	0.0168	6.1
hsa-miR-662	0.0009	0.0146	104.5	hsa-miR-505-3p	0.0026	0.0168	6.4
hsa-miR-99b-3p	0.0009	0.0146	109.4	hsa-miR-150-5p	0.0036	0.0191	3.1
hsa-miR-H1-5p	0.0009	0.0146	4.6	hsa-miR-186-5p	0.0036	0.0191	27.1
hsa-miR-4322	0.0012	0.0167	7.7	hsa-miR-28-5p	0.0036	0.0191	19.6
hsa-miR-516a-5p	0.0012	0.0167	68.5	hsa-miR-130a-3p	0.0050	0.0223	6.6
hsa-miR-3682-3p	0.0015	0.0167	3.1	hsa-miR-185-5p	0.0050	0.0223	23.2
hsa-miR-1226-5p	0.0018	0.0167	16.1	hsa-miR-361-3p	0.0050	0.0223	39.6
hsa-miR-610	0.0018	0.0167	59.6	hsa-miR-374c-5p	0.0070	0.0283	21.4
hsa-miR-K12-8-5p	0.0021	0.0168	7.7	hsa-miR-128	0.0095	0.0361	21.3
hsa-miR-3937	0.0026	0.0168	13.8	hsa-miR-214-3p	0.0128	0.0462	8.3
hsa-miR-1273d	0.0030	0.0190	8.5	hsa-miR-146b-5p	0.0148	0.0509	4.7
hsa-miR-3132	0.0030	0.0190	3.4	hsa-miR-548c-3p	0.0148	0.0509	5.4
hsa-miR-3147	0.0036	0.0191	17.4	hsa-miR-100-5p	0.0172	0.0575	14.8
hsa-miR-3654	0.0036	0.0191	5.0	hsa-miR-652-3p	0.0229	0.0701	12.7
hsa-miR-1273c	0.0042	0.0207	5.8	hsa-miR-139-3p	0.0299	0.0842	4.4
hsa-miR-3174	0.0042	0.0207	8.2	hsa-miR-130b-3p	0.0387	0.0949	3.5
hsa-miR-3622b-5p	0.0070	0.0283	6.4	hsa-miR-192-3p	0.0389	0.0949	15.9
hsa-miR-628-3p	0.0095	0.0361	11.3	hsa-miR-30a-5p	0.0389	0.0949	6.9
hsa-miR-601	0.0110	0.0405	7.9	hsa-miR-365a-3p	0.0389	0.0949	3.9
hsa-miR-370	0.0148	0.0509	6.0	hsa-miR-101-3p	0.0390	0.0949	7.1
hsa-miR-548q	0.0172	0.0575	15.0	hsa-miR-195-5p	0.0390	0.0949	4.6
hsa-miR-1287	0.0198	0.0641	16.8	hsa-miR-199b-5p	0.0390	0.0949	7.0
hsa-miR-149-3p	0.0229	0.0701	10.3	hsa-miR-29b-1-5p	0.0390	0.0949	4.5
hsa-miR-584-5p	0.0261	0.0775	11.9				
hsa-miR-B2RC	0.0299	0.0842	6.6				
hsa-miR-887	0.0299	0.0842	14.8				
hsa-miR-3622a-5p	0.0300	0.0842	7.2				
hsa-miR-4261	0.0300	0.0842	7.7				
hsa-miR-4253	0.0390	0.0949	16.7				

FDR, false discovery rate.
