# Supplementary material for: Predictive microRNAs for lymph node metastasis in endoscopically resectable submucosal colorectal cancer
Source: Oncotarget. 2016 Apr 16;7(22):32902–15. doi: 10.18632/oncotarget.8766 (PMC5078061; doi:10.18632/oncotarget.8766)
Supplement: Supplementary file 3 [file oncotarget-07-32902-s003.pdf]

Table S3. Selection and replication of the 13 miRNAs

miRNA	Ref	Event*	Classifier construction set-I (microarray)#			Classifier construction set-I (qPCR)			Classifier construction set-II (qPCR)			Overall (qPCR)			
			LNM + (average)	LNM- (average)	*P*-value	LNM+ (average)	LNM- (average)	*P*-value	LNM+ (average)	LNM- (average)	*P*-value	LNM+ (average)	LNM- (average)	Fold Change	*P*-value
miR-342-3p	[1]	Down	-3.2036	0.5389	0.0009	0.3999	0.6678	0.0340	0.4786	0.7834	0.0030	0.4442	0.7313	1.6	$4.3x10^{-4}$
miR-195-5p	[2]	Down	-2.0273	0.1801	0.0390	0.2043	0.3414	0.1530	0.1613	0.3252	0.0020	0.1801	0.2830	1.6	0.0020
miR-150-5p	[3]	Down	-0.9272	0.6937	0.0036	0.1818	0.4389	0.0640	0.2613	0.6218	0.0140	0.2265	0.5395	2.4	0.0030
miR-140-3p	[4]	Down	-2.2145	0.2728	0.0018	0.4442	0.5358	0.0300	0.4330	0.6873	0.0300	0.4379	0.6191	1.4	0.0046
miR-361-3p	[5]	Down	-0.4077	4.9006	0.0050	0.4132	0.7462	0.0100	0.6308	0.8023	0.1190	0.5356	0.7771	1.5	0.0046
miR-192-3p	[6]	Down	-3.6052	0.3842	0.0389	0.3454	0.5983	0.0640	0.3734	0.6322	0.0440	0.3611	0.6170	1.7	0.0051
miR-200b-5p	[7]	Down	-2.3314	0.2726	0.0025	0.5950	1.1286	0.0100	0.7259	0.7463	0.4700	0.6686	0.9183	1.4	0.0280
miR-185-5p	[8]	Down	-2.7170	1.8179	0.0050	0.5463	0.7794	0.1860	0.7428	1.0928	0.0370	0.6569	0.9518	1.4	0.0280
miR-30a-5p	[9]	Down	-2.8202	-0.0332	0.0389	0.5579	0.5327	0.1010	0.4301	0.4889	0.3820	0.4860	0.5086	1.0	0.0520
miR-28-5p	[10]	Down	-3.7159	0.5783	0.0036	0.4561	0.5215	0.2660	0.4804	0.5980	0.0740	0.4697	0.5636	1.2	0.0860
miR-3621	[11]	Up	5.2540	0.0445	0.0009	1.4926	0.2572	0.0008	0.9607	0.7563	0.9090	1.1934	0.5317	2.2	0.0040
miR-1287	[12]	Up	0.8249	-3.2437	0.0198	0.6068	0.3212	0.1010	0.6700	0.3627	0.0530	0.6424	0.3440	1.9	0.0060
miR-3132	[13]	Up	0.4322	-1.3375	0.0030	7.8693	1.6593	0.0170	2.6376	2.0670	0.3820	4.9265	1.8835	2.6	0.0200

Classifier construction set-I: 16 T1-stage CRCs (7 LNM-positive and 9 LNM-negative tumors)
Classifier construction set-II: 20 T1-stage CRCs (9 LNM-positive and 11 LNM-negative tumors)
Overall: 36 T1-stage CRCs (16 LNM-positive and 20 LNM-negative tumors
*Down, relatively downregulated (LNM+/LNM-); Up, relatively upregulated (LNM+/LNM-)
#The average expression levels are log2 scale.
LNM, lymph node metastasis
