# Supplementary material for: Predictive microRNAs for lymph node metastasis in endoscopically resectable submucosal colorectal cancer
Source: Oncotarget. 2016 Apr 16;7(22):32902–15. doi: 10.18632/oncotarget.8766 (PMC5078061; doi:10.18632/oncotarget.8766)
Supplement: Supplementary file 4 [file oncotarget-07-32902-s004.pdf]

**Table S5.** Top twenty pathways significantly enriched with the predicted target genes of the three miRNAs.

| miRNA | PathName | PathFg* | PathBg** | pval | BH |
|---|---|---|---|---|---|
| hsa-miR-342-3p | Insulin signaling pathway | 90 | 139 | 2.59E-08 | 5.00E-06 |
| hsa-miR-342-3p | Neurotrophin signaling pathway | 84 | 129 | 5.11E-08 | 9.80E-06 |
| hsa-miR-342-3p | Pathways in cancer | 184 | 330 | 1.06E-07 | 2.02E-05 |
| hsa-miR-342-3p | Axon guidance | 83 | 129 | 1.35E-07 | 2.55E-05 |
| hsa-miR-342-3p | Long term potentiation | 51 | 71 | 2.19E-07 | 4.14E-05 |
| hsa-miR-342-3p | Renal cell carcinoma | 51 | 71 | 2.19E-07 | 4.14E-05 |
| hsa-miR-342-3p | Focal adhesion | 118 | 203 | 1.24E-06 | 0.000229 |
| hsa-miR-342-3p | Glioma | 46 | 65 | 1.70E-06 | 0.000315 |
| hsa-miR-342-3p | Adherens junction | 52 | 76 | 1.91E-06 | 0.000351 |
| hsa-miR-342-3p | Phosphatidylinositol signaling system | 52 | 76 | 1.91E-06 | 0.000351 |
| hsa-miR-342-3p | Wnt signaling pathway | 91 | 152 | 3.67E-06 | 0.000676 |
| hsa-miR-342-3p | Colorectal cancer | 56 | 86 | 8.34E-06 | 0.001509 |
| hsa-miR-342-3p | Inositol phosphate metabolism | 38 | 54 | 1.68E-05 | 0.002986 |
| hsa-miR-342-3p | Non small cell lung cancer | 38 | 54 | 1.68E-05 | 0.002986 |
| hsa-miR-342-3p | Regulation of actin cytoskeleton | 118 | 212 | 2.13E-05 | 0.003766 |
| hsa-miR-342-3p | MAPK signaling pathway | 146 | 272 | 3.16E-05 | 0.005568 |
| hsa-miR-342-3p | ErbB signaling pathway | 56 | 89 | 3.69E-05 | 0.006461 |
| hsa-miR-342-3p | TGF beta signaling pathway | 54 | 86 | 5.49E-05 | 0.009556 |
| hsa-miR-342-3p | Endocytosis | 104 | 187 | 6.65E-05 | 0.011502 |
| hsa-miR-342-3p | Chronic myeloid leukemia | 48 | 75 | 6.99E-05 | 0.012099 |
| hsa-miR-361-3p | MAPK signaling pathway | 153 | 272 | 7.94E-11 | 1.52E-08 |
| hsa-miR-361-3p | Pathways in cancer | 179 | 330 | 1.23E-10 | 2.35E-08 |
| hsa-miR-361-3p | Colorectal cancer | 59 | 86 | 2.79E-09 | 5.27E-07 |
| hsa-miR-361-3p | Wnt signaling pathway | 92 | 152 | 3.39E-09 | 6.41E-07 |
| hsa-miR-361-3p | Insulin signaling pathway | 83 | 139 | 4.64E-08 | 8.73E-06 |
| hsa-miR-361-3p | Pancreatic cancer | 50 | 75 | 1.85E-07 | 3.41E-05 |
| hsa-miR-361-3p | VEGF signaling pathway | 51 | 78 | 3.54E-07 | 6.55E-05 |
| hsa-miR-361-3p | Axon guidance | 76 | 129 | 3.63E-07 | 6.72E-05 |
| hsa-miR-361-3p | Hedgehog signaling pathway | 39 | 56 | 7.43E-07 | 0.000136 |
| hsa-miR-361-3p | Non small cell lung cancer | 37 | 54 | 2.66E-06 | 0.00047 |
| hsa-miR-361-3p | Acute myeloid leukemia | 39 | 58 | 2.96E-06 | 0.000522 |
| hsa-miR-361-3p | Neurotrophin signaling pathway | 73 | 129 | 4.89E-06 | 0.000846 |
| hsa-miR-361-3p | mTOR signaling pathway | 36 | 53 | 4.97E-06 | 0.000856 |
| hsa-miR-361-3p | ErbB signaling pathway | 54 | 89 | 5.08E-06 | 0.000873 |
| hsa-miR-361-3p | Prostate cancer | 54 | 89 | 5.08E-06 | 0.000873 |
| hsa-miR-361-3p | Glioma | 42 | 65 | 5.95E-06 | 0.001021 |
| hsa-miR-361-3p | Renal cell carcinoma | 45 | 71 | 5.99E-06 | 0.001025 |
| hsa-miR-361-3p | Endometrial cancer | 35 | 52 | 9.21E-06 | 0.001556 |
| hsa-miR-361-3p | Calcium signaling pathway | 94 | 178 | 1.27E-05 | 0.002122 |
| hsa-miR-361-3p | Chronic myeloid leukemia | 46 | 75 | 1.68E-05 | 0.002784 |
| hsa-miR-3621 | Pathways in cancer | 214 | 330 | 3.99E-11 | 7.69E-09 |
| hsa-miR-3621 | Wnt signaling pathway | 111 | 152 | 5.95E-11 | 1.15E-08 |
| hsa-miR-3621 | Endocytosis | 127 | 187 | 5.78E-09 | 1.11E-06 |
| hsa-miR-3621 | Chronic myeloid leukemia | 59 | 75 | 1.92E-08 | 3.64E-06 |
| hsa-miR-3621 | MAPK signaling pathway | 173 | 272 | 2.36E-08 | 4.49E-06 |
| hsa-miR-3621 | Axon guidance | 91 | 129 | 5.10E-08 | 9.62E-06 |
| hsa-miR-3621 | Acute myeloid leukemia | 47 | 58 | 1.02E-07 | 1.90E-05 |
| hsa-miR-3621 | Adherens junction | 58 | 76 | 1.76E-07 | 3.27E-05 |
| hsa-miR-3621 | Pancreatic cancer | 57 | 75 | 2.88E-07 | 5.33E-05 |
| hsa-miR-3621 | ErbB signaling pathway | 65 | 89 | 5.45E-07 | 0.000101 |
| hsa-miR-3621 | Colorectal cancer | 61 | 86 | 5.98E-06 | 0.001064 |
| hsa-miR-3621 | Glioma | 48 | 65 | 9.98E-06 | 0.001747 |
| hsa-miR-3621 | Apoptosis | 61 | 87 | 1.08E-05 | 0.001896 |
| hsa-miR-3621 | Neurotrophin signaling pathway | 85 | 129 | 1.16E-05 | 0.002024 |
| hsa-miR-3621 | Prostate cancer | 62 | 89 | 1.28E-05 | 0.002225 |

| | | | | | |
|---|---|---|---|---|---|
| hsa-miR-3621 | Insulin signaling pathway | 90 | 139 | 1.86E-05 | 0.003203 |
| hsa-miR-3621 | Phosphatidylinositol signaling system | 54 | 76 | 1.87E-05 | 0.003203 |
| hsa-miR-3621 | Long term potentiation | 51 | 71 | 1.95E-05 | 0.003335 |
| hsa-miR-3621 | Melanogenesis | 69 | 102 | 2.04E-05 | 0.003496 |
| hsa-miR-3621 | Basal cell carcinoma | 41 | 55 | 3.04E-05 | 0.005106 |

* PathFg stand for number of genes predicted as putative targets in a given pathway.

** PathBg stand for number of genes in a given pathway.
