# Supplementary material for: Predictive microRNAs for lymph node metastasis in endoscopically resectable submucosal colorectal cancer
Source: Oncotarget. 2016 Apr 16;7(22):32902–15. doi: 10.18632/oncotarget.8766 (PMC5078061; doi:10.18632/oncotarget.8766)
Supplement: Supplementary file 5 [file oncotarget-07-32902-s005.pdf]

Table S6. Top twenty gene ontology sets significantly enriched with the predicted target genes of the three miRNAs.

miRNA	Category[#]	PathName	PathFg*	PathBg**	pval	BH
hsa-miR-342-3p	MF	zinc ion binding	992	1950	1.17E-18	1.96E-16
hsa-miR-342-3p	BP	transcription dna dependent	898	1765	6.48E-17	2.85E-14
hsa-miR-342-3p	MF	dna binding	807	1612	3.37E-13	5.59E-11
hsa-miR-342-3p	BP	regulation of transcription dna dependent	630	1221	1.56E-13	6.83E-11
hsa-miR-342-3p	CC	golgi apparatus	348	643	4.19E-11	5.03E-09
hsa-miR-342-3p	BP	nerve growth factor receptor signaling pathway	138	218	6.12E-11	2.68E-08
hsa-miR-342-3p	BP	signal transduction	507	987	1.02E-10	4.47E-08
hsa-miR-342-3p	CC	Cytoplasm	1812	3964	1.37E-09	1.63E-07
hsa-miR-342-3p	MF	sequence specific dna binding transcription factor activity	453	884	1.59E-09	2.63E-07
hsa-miR-342-3p	BP	positive regulation of transcription dna dependent	264	481	1.60E-09	6.97E-07
hsa-miR-342-3p	MF	atp binding	713	1470	1.17E-08	1.92E-06
hsa-miR-342-3p	BP	axon guidance	180	312	5.01E-09	2.18E-06
hsa-miR-342-3p	MF	protein serine threonine kinase activity	182	320	1.79E-08	2.92E-06
hsa-miR-342-3p	CC	cell junction	189	337	4.03E-08	4.75E-06
hsa-miR-342-3p	MF	protein kinase binding	152	262	4.64E-08	7.52E-06
hsa-miR-342-3p	MF	transcription factor binding	154	268	8.93E-08	1.44E-05
hsa-miR-342-3p	CC	integral to plasma membrane	509	1033	1.47E-07	1.72E-05
hsa-miR-342-3p	BP	synaptic transmission	209	382	1.09E-07	4.71E-05
hsa-miR-342-3p	BP	protein phosphorylation	188	340	1.73E-07	7.49E-05
hsa-miR-342-3p	BP	epidermal growth factor receptor signaling pathway	83	130	2.21E-07	9.56E-05
hsa-miR-361-3p	CC	cytoplasm	1678	3964	1.57E-14	1.72E-12
hsa-miR-361-3p	MF	sequence specific dna binding transcription factor activity	426	884	3.33E-12	5.26E-10
hsa-miR-361-3p	CC	cell junction	183	337	6.78E-11	7.39E-09
hsa-miR-361-3p	CC	integral to plasma membrane	481	1033	9.37E-11	1.01E-08
hsa-miR-361-3p	BP	positive regulation of transcription from rna polymerase ii promoter	316	636	2.96E-11	1.30E-08
hsa-miR-361-3p	MF	zinc ion binding	852	1950	1.57E-10	2.46E-08
hsa-miR-361-3p	CC	nucleus	1943	4759	4.93E-10	5.28E-08
hsa-miR-361-3p	MF	phospholipid binding	167	308	5.31E-10	8.29E-08
hsa-miR-361-3p	BP	positive regulation of transcription dna dependent	241	481	2.53E-09	1.11E-06
hsa-miR-361-3p	BP	signal transduction	453	987	3.65E-09	1.59E-06
hsa-miR-361-3p	BP	axon guidance	166	312	3.79E-09	1.65E-06
hsa-miR-361-3p	MF	transcription regulatory region dna binding	93	158	2.00E-08	3.11E-06
hsa-miR-361-3p	BP	intracellular signal transduction	135	247	1.21E-08	5.23E-06
hsa-miR-361-3p	BP	protein autophosphorylation	91	153	1.40E-08	6.05E-06
hsa-miR-361-3p	CC	cytosol	977	2321	6.78E-08	7.19E-06
hsa-miR-361-3p	BP	nerve growth factor receptor signaling pathway	121	218	2.09E-08	9.02E-06

hsa-miR-361-3p	MF	protein serine threonine kinase activity	165	320	7.27E-08	1.12E-05
hsa-miR-361-3p	MF	sequence specific dna binding	241	498	1.15E-07	1.76E-05
hsa-miR-361-3p	CC	golgi apparatus	301	643	1.80E-07	1.89E-05
hsa-miR-361-3p	BP	transcription dna dependent	759	1765	4.80E-08	2.07E-05
hsa-miR-3621	MF	protein binding	2742	4920	3.04E-45	5.59E-43
hsa-miR-3621	CC	nucleus	2521	4759	3.24E-21	4.83E-19
hsa-miR-3621	CC	cytoplasm	2120	3964	6.74E-20	9.98E-18
hsa-miR-3621	MF	atp binding	837	1470	1.80E-15	3.30E-13
hsa-miR-3621	CC	cytosol	1252	2321	8.44E-13	1.24E-10
hsa-miR-3621	BP	axon guidance	209	312	6.50E-13	3.56E-10
hsa-miR-3621	BP	positive regulation of transcription dna dependent	302	481	1.59E-12	8.68E-10
hsa-miR-3621	CC	integral to plasma membrane	584	1033	2.18E-10	3.18E-08
hsa-miR-3621	BP	positive regulation of transcription from rna polymerase ii promoter	378	636	1.28E-10	6.96E-08
hsa-miR-3621	BP	nerve growth factor receptor signaling pathway	149	218	1.39E-10	7.58E-08
hsa-miR-3621	CC	nucleolus	830	1525	1.12E-09	1.63E-07
hsa-miR-3621	BP	nervous system development	188	290	6.26E-10	3.40E-07
hsa-miR-3621	CC	cell junction	212	337	2.59E-09	3.73E-07
hsa-miR-3621	BP	transmembrane transport	276	451	7.24E-10	3.93E-07
hsa-miR-3621	CC	golgi membrane	274	452	3.20E-09	4.58E-07
hsa-miR-3621	CC	membrane	409	711	7.52E-09	1.07E-06
hsa-miR-3621	BP	wnt receptor signaling pathway	99	138	3.17E-09	1.72E-06
hsa-miR-3621	BP	negative regulation of transcription from rna polymerase ii promoter	275	457	8.24E-09	4.46E-06
hsa-miR-3621	CC	synapse	77	105	3.71E-08	5.24E-06
hsa-miR-3621	BP	signal transduction	550	987	1.29E-08	6.98E-06

# BP, biological process; CC, cellular component; MF, molecular function.

* PathFg stand for number of genes predicted as putative targets in a given pathway.

** PathBg stand for number of genes in a given pathway.
